# NIST/Sandia/ICDD Electron Diffraction Database: A Database for Phase Identification by Electron Diffraction

**DOI:** 10.6028/jres.094.003

**Published:** 1989

**Authors:** M. J. Carr, W. F. Chambers, D. Melgaard, V. L. Himes, J. K. Stalick, A. D. Mighell

**Affiliations:** Sandia National Laboratories Albuquerque, NM 87185; J&M Systems Albuquerque, NM 87123; National Institute of Standards and Technology Gaithersburg, MD 20899

**Keywords:** electron diffraction, identification, numeric database, phase characterization

## Abstract

A new database containing crystallographic and chemical information designed especially for application to electron diffraction search/match and related problems has been developed. The new database was derived from two well-established x-ray diffraction databases, the JCPDS Powder Diffraction File and NBS CRYSTAL DATA, and incorporates 2 years of experience with an earlier version. It contains 71,142 entries, with space group and unit cell data for 59,612 of those. Unit cell and space group information were used, where available, to calculate patterns consisting of all allowed reflections with *d*-spacings greater than 0.8 A for ~ 59,000 of the entries. Calculated patterns are used in the database in preference to experimental x-ray data when both are available, since experimental x-ray data sometimes omits high *d*-spacing data which falls at low diffraction angles. Intensity data are not given when calculated spacings are used. A search scheme using chemistry and *r*-spacing (reciprocal *d*-spacing) has been developed. Other potentially searchable data in this new database include space group, Pearson symbol, unit cell edge lengths, reduced cell edge length, and reduced cell volume. Compound and/or mineral names, formulas, and journal references are included in the output, as well as pointers to corresponding entries in NBS CRYSTAL DATA and the Powder Diffraction File where more complete information may be obtained. Atom positions are not given. Rudimentary search software has been written to implement a chemistry and *r*-spacing bit map search. With typical data, a full search through ~ 71,000 compounds takes 10~20 seconds on a PDP 11/23-RL02 system.

## Introduction

The identification of Ciystalline objects in the size range from 10 *μ*m to 10 Å is readily accomplished in the analytical electron microscope (AEM) if the analyst has access to appropriate information. Most often the needed information exsists, but either it is not readily accessible in the laboratory or it is not in the most useful form. Acquiring and reprocessing reference data is often the time-limiting step in the identification process. Information scattered through the open literature has been collected into compilations which recently have become available in computer-readable form [1,2]. Even so, the format of the data is not ideally suited for electron diffraction work [3].

We perceived a need for a specialized database to support efficient phase identification by combined electron diffraction and energy dispersive x-ray spectroscopy (EDS) in a modern analytical electron microscope. Considering the quality of the experimental data obtainable from the AEM, the quantity of reference data, and available computing machinery, we set out to create a database to support search/match procedures [4] and crystallographic calculations [5] performed routinely in our laboratories.

## Description of the Database

This database was derived from two copyrighted databases, NBS CRYSTAL DATA and the PDF-2. The preparation of the derivative database was facilitated by the fact that the original databases are in the same format since both were built with a program called NBS*AIDS83 [6]. The new derivative database contains a subset of information from the full databases, selected on the basis of pertinence to electron diffraction analysis. Only inorganic compounds were used [7]. The data is accurate and as complete as possible, but has been reduced in precision to a level appropriate for electron diffraction work (±~1%@1.5 Å). It has been packed in a manner which allows it to be used on a small computer equipped with a 10 Mb hard disk. The database is complete so that it is useful without reference to other sources such as cards [I] or books [1,2], but it contains pointers so that if a card file [1], CDROM [8], or other full listing [1,2] is available, one can quickly get to that information as well.

The data were selected from the two sources as follows:

1. All inorganic compounds from NBS CRYSTAL DATA were used. The unit cell and space group information from each compound were used to compute up to 60 non-redundant allowed reflections with *d*-spacings greater than 0.8 Å. Intensities were not computed. There are 59,612 entries of this type.
2. Inorganic compounds from PDF-2 sets 1–33 whose entries do not give unit cell data, and all entries from sets 34–36 were used. These are only a subset of the full PDF-2 database. It was assumed that entries having unit cell information in sets 1–33 are adequately represented by similar entries in NBS CRYSTAL DATA and would only duplicate information. *d*-spacings and intensities (obtained from x-ray methods) were used. All inorganic compounds from PDF-2 sets 34–36 were used whether or not they contained unit cell information, since it could not be assessed whether such compounds had been included in NBS CRYSTAL DATA yet. (A little duplication is better than missing a compound altogether.) This group contains 11,530 entries.

Despite their different origins, the two types of source data are functionally equivalent and are treated equally in the new database. They are mingled in the ordered and indexed Search file. The computed data (1.) represents the best target group for matching on the basis of observed *d*-spacings from single diffraction patterns. The data in group (2.) is similar to the data obtainable from the PDF Level I database, an earlier version of the PDF-2 used in this work. We have searched against type (2.) data for over two years with fair success [3]. When searching failed, it was often because the experimental x-ray observations in the PDF Level I database did not include high *d*-spacing reflections observable by electron diffraction. The computed data in (1.) is an attempt to correct this weakness, but computation is not possible for compounds in (2.) because unit cell and space group information is absent. The data in (2.) is valuable nonetheless, because even if you cannot completely characterize such a material, at least you can determine that “you found it again.” The literature reference may be of some use in such cases.

As in the earlier version of this work, data are stored in two types of files: Reference files and a Search file. We have kept sufficient information in each entry to be of use for electron diffraction analysis, but have put only certain critical information in the Search file, for the sake of speed. The data for each compound, therefore, is divided between the Search file and a Reference file. There may be more than one entry for a given compound. Multiple entries for the same compound are present mainly when derived from different literature citations.

The contents of a Reference file entry for a given compound are:

**Table:** 

1. Name length (1 byte)	Number of bytes (x) to store the compound name.
2. Formula length (I byte)	Number of bytes (y) to store the compound formula.
3. # of intensities (1 byte)	Number of reflections (z) having intensities (if computed, then 0).
4. Unit cell angles (4 bytes)	Number and kind of angles given for the conventional unit cell.
5. Reduced cell angles (4 bytes)	Number and kind of angles given for the reduced cell [4].
6. Pearson symbol (4 bytes)	xXnnnn, indicates crystal class, symmetry, and number of atoms in the conventional unit cell.
7. Journal reference (17 bytes)	CODEN, volume, page, and first 9 characters of the author name field (Radix-50), and year (−1800).
8. Source ID (3 bytes)	PDF number or CRYSTAL DATA ID number.
9. Unit cell angles (0–12 bytes)	Degrees* 100, only the necessary ones.
10. Reduced cell angles (0–12 bytes)	Degrees* 100, only the necessary ones.
11. Compound name (x bytes)	Including mineral name, if applicable (Radix 50).
12. Compound formula (y bytes)	Functional formula (ASCII).
13. Intensitites (>z/2bytes)	In nibbles, if present (a nibble=4 bits =1/2 a byte) always ending on a word boundary

The first eight items are fixed length fields; the last five vary in length and may be absent. The entries in the Reference files therefore vary in length. Angles are multiplied by 100 and rounded to convert them to integers, which take less storage than floating point numbers while preserving two decimal place precision. Only angles not equal to 90 degrees are stored, with a code indicating whether they represent *α*, *β*, or *γ.* Missing angles are always 90°. The compound names are converted to Radix-50 notation which encodes 3 characters per 2 bytes (50% denser than packed character strings). Reference entries are grouped together in 16 Reference files, each of which contain a large number (2000–5000) entries. Twelve reference files contain data from NBS CRYSTAL DATA entries, and four files contain data from PDF-2 compounds. There is a pointer to the corresponding Reference file entry stored in each Search file entry. The Reference files are not meant to be searched, but rather to be directly accessed one time, after a search has been completed. In total, these files require ~4.5 Mb of disk.

The contents of a Search file entry for a given compound are:

**Table:** 

1. Chemistry bit map (12 bytes);	Elements 1–96 in six 16-bit words.
2. r-spacing bit map (22 bytes):	Eleven 16-bit words, representing 176 cells, each 0.018 Å wide, of *r*-spacing (*r* = λ*L*/*d*, where λL =2.5 Å-cm). At λL = 2.5 Å-cm, *r*-spacings range from 0.0 to 3.2 cm, representing *d*-spacings from ∞ to 0.8 Å).
3. Space group (2 bytes):	Encoded in two bytes, allows for *nnnX *, if present, signifies that the space group is not completely determined, so an aspect is given [6]. nnn is the space group number (1–230). X, if present, gives the setting, e.g., Pbca or Pcab, etc.
4. Unit cell edges (6 bytes):	Å* 100, three 16-bit integers, the dimensions of the unit cell given in NBS CRYSTAL DATA, which may be different from the unit cell assigned by the original author.
5. Reduced cell edges (6 bytes):	A* 100, three 16-bit integers, the dimensions of a mathematically unique primitive unit cell equivalent to the conventional unit cell.
6. Reduced cell volume (2 bytes):	Used by the NBS Lattice search program.
7. Flags (8 bits):	Organic/inorganic, mineral, metals & alloys, hydrate, deleted, NHx-containing, unit cell differs from original author’s cell, and a spare.
8. Pointer (3 bytes):	To the corresponding Reference file entry.
9. Spare word (2 bytes):	In case something simple needs to be added in the future.

This is a single large file (~4 Mb). The entries are ordered on the basis of composition, beginning with atomic number 11 (sodium). We have assumed an EDS detector with a beryllium window is being used. This is the most common type of detector in the field today. It is capable of detecting only elements whose characteristic x rays are hard enough to penetrate the Be window (namely *Z*⩾ 11). This orders the file on the basis of EDS-detectable qualitative chemistry, scattering oxides, carbides, etc., through the file associated with their EDS-observable elements. This ordering is advantageous even when using an EDS detector that can detect lighter elements, because the light elements are so common in compounds in the file as to be a disadvantage when searching. For example, oxygen is present in more than half of all the compounds in the file, so it is much more efficient to go looking for iron-bearing compounds (5909) that contain oxygen (3837), than oxygen-bearing compounds (40084) that contain iron (3837). The ordering scheme also places compounds containing only undetectable light elements (e.g., ice, graphite, boron nitride) at the end of the file, where they may be skipped as a group if so desired. Each entry in this file is a fixed length (56 bytes). Entries are grouped into records. There are 18 entries in each record, followed by 16 empty bytes to pad the record length to 1024 bytes (two blocks). This facilitates a speedy search by creating a constant off-set or spacing between fields of the same type within a record, and allows for easy disk access with a two-block buffer.

The first part of the Search file contains an index to the records in the remainder of the Search file. The indexing scheme was described in detail previously [3].

There is one index entry for each record in the Search file. The Index file is 60 Kb in size. There are 18 compounds per 1024-byte record in the Search file, so each entry in this index file refers to 18 compounds. Because the Search file is ordered by chemisty, the Index file makes it possible to perform a coarse screening (in groups of 18) of the Search file to find the records which may contain compounds with the proper chemistry. More directly, the index allows the search software to apply a quick test and then most often skip over a group of compounds which certainly contain no possible matches based on the chemistry requirements. This greatly reduces the number of Search file entries which must be processed in detail and can increase the overall speed of the search by as much as an order of magnitude.

The structure of the file is based on our search/match experience with an earlier version of this database. It is designed to be searched first on the basis of chemistry, which has been shown to be the primary characteristic in electron diffraction phase identification work [9]. The index file allows one to skip over large sections of the file where no chemistry matches are possible, greatly reducing search time. After considering chemistry, we can perform a secondary match on the basis of observed *r*-spacings, or on the basis of flags indicating membership in one or more subsets of the data. It is also possible to make no requirements on chemistry, in which case all entries in the file will pass the chemistry test. Then, a search takes the maximum amount of time since every entry will be tested for secondary match requirements. It is possible to search on the basis of other parameters, such as space group, Pearson Symbol, reduced cell parameters [4], or unit cell parameters, although we have not developed software to do so. Since the unit cell parameters are stored for most of the compounds, it is possible to write additional software to quickly calculate precise *d*-spacings and Miller indices of allowed reflections for a particular compound if the need arises.

The contents of an Index entry for a given compound are:

**Table:** 

1. Bnum:	Block number in the Search file (2 bytes).
2. ORmap:	Six 16-bit words containing the result of performing the boolean OR function on the chemistry bit maps for all the compounds in one record.

## Search/Match Software

Source code for basic functional search/match software is distributed with the database. Two versions exist. An assembly language search algorithm was written and described for the first generation of this file [3]. The general nature of the algorithm remains the same for this file, with minor changes to accommodate the format of the new database. Experience with typical data (2 or 3 observed elements, all unobserved heavy elements and some light elements excluded, 6–8 diffraction spots) has shown that most searches require 10–20 seconds to search the full file on a PDF 11/23 equipped with an RL02 10 Mbyte hard disk; I/O takes several times longer than that. It is also possible to write search programs for this file in high level languages. FORTRAN versions have implemented the same search on VAX, PDF, and SUN computers. On the FDP, the FORTRAN version gives the same results but runs five times slower than the assembly language version. Similar programs could be written in other languages that support bit manipulation. A version of this software has been written in Flextran to be integrated into the RAD group of programs [5] which run on computerized EDS analysis equipment attached to an electron microscope. Users are encouraged to modify or add to the programs. Additional software for searching on the basis of reduced cell [4] or space group may be added at a later date.

## Conclusion

The database described in this report contains what we believe to be the only complete collection of inorganic compound data structured for phase identification by electron diffraction available. Nevertheless, the database is small enough to reside on a personal computer or laboratory microcomputer dedicated to EDS analysis in an electron microscope laboratory.

Since the database was designed especially for electron diffraction analysis, it is not expected to work well for traditional x-ray diffraction analysis where high precision data for both peak position and intensity are obtained and used.

It is anticipated that many different search/match schemes will be able to use this database, although we have initially implemented only one. Searching first on the basis of qualitative EDS chemistry is a natural consequence of the type of information obtained with the AEM and greatly increases searching speed in a small computer. The computed data, incorporating high *d*-spacing reflections, are very diagnostic for electron diffraction search/match identification. Beyond its usefulness as a search/match tool, the database also provides a convenient resource for crystallographic data for pattern simulation. The full integration of this database into our existing analytical software is planned, and we expect that it will be useful to other laboratories as well.

The development of this database has been a joint project at Sandia National Laboratories and the National Institute of Standards and Technology, with the encouragement of the JCPDS/ICDD. Further evolution of this database and any related items will be guided by the members of the Phase Identification by Electron Diffraction subcommittee of the JCPDS Technical Committee and the NIST Crystal Data Center. The details of the database format, software to generate and update the database from original source tapes, and the search/match software are available upon request. The database itself is copyrighted by the National Institute of Standards and Technology and is being distributed by license through the JCPDS/ICDD. For information on obtaining the database contact JCPDS/ICDD headquarters [1] or the NIST Crystal Data Center [2].

## References

[b1-jresv94n1p15_a1b] 1PDF-2. The master database of chemical, crystallographic, and x-ray powder diffraction data compiled and evaluated by the Joint Committee on Powder Diffraction Standards, International Centre for Diffraction Data, 1601 Park Lane, Swarthraore, PA, 19081. The PDF-2 database contains all the information on the familiar PDF cards and is available on cards and magnetic tape by license only.

[b2-jresv94n1p15_a1b] 2NBS CRYSTAL DATA (1987). The master database of chemical and crystallographic data compiled and evaluated by the NIST Crystal Data Center, National Institute of Standards and Technology Gaithersburg, MD 20899. The full database is available on magnetic tape by license only. Portions of the data are available in book form as Crystal Data Determinative Tables in several volumes.

[3] Carr MJ, Chambers WF, Melgaard DA (1986). Search/Match Procedure for Electron Diffraction Data Based on Pattern Matching in Binary Bit Maps. Powder Diffraction.

[4] Himes VL, Mighell AD (1985). NBS*LATTICE, A Program to Analyze Lattice Relationships.

[5] Carr MJ, Chambers WF (1984). A Review of Crystallographic Calculational Methods used in the RAD Group of Computer Programs for Analytical Electron Microscopy. J Microsc.

[b6-jresv94n1p15_a1b] 6The NBS*AIDS83 data evaluation and database-building computer program was developed at the National Institute of Standards and Technology for use by both the JCPDS/ICDD and the NIST Crystal Data Center. Two distribution databases were created and are maintained with this program—NBS CRYSTAL DATA and JCPDS/ICDD PDF-2. The research uses of this program have been described earlier (Mighell, A. D., Hubbard, C. R., and Stalick, J. K., NBS*AIDS80: A FORTRAN Program for Crystallographic Data Evaluation, NBS Technical Note 1141, 1981). The user is referred to a description of NBS CRYSTAL DATA for detailed information on many items in common to both databases (Stalick, J. K., and Mighell, A. D., CRYSTAL DATA. Version 1.0 Database Specifications, NBS Technical Note 1229, 1986). The program as implemented by the JCPDS/ICDD contains many codes and conventions specific to the PDF-2 database and descriptions of these items are included in a program user’s manual which is available from the JCPDS/ICDD or the NIST Crystal Data Center.

[b7-jresv94n1p15_a1b] 7Organic compounds, representing more than 80,000 additional entries, were not included because, in general, they are sensitive to degradation in the electron-beam/high-vacuum environment and are rarely successfully analyzed by AEM. On a day-to-day basis in most materials science laboratories where this database is likely to be used, these compounds would only occupy disk space and increase search times. If a need arises in the future, these compounds could be added to this database with no modification of its basic structure. Some compounds with organic components, flagged as inorganic in the original databases, are included in the new database.

[b8-jresv94n1p15_a1b] 8The PDF-2 and NBS CRYSTAL DATA, which in full AIDS format occupy ~250 Mbyte of storage, have recently become available on an optical, read-only mass storage device called a CDROM, which is similar to CDs available for home audio systems. A drive which reads the CDROM is available for IBM and compatible PCs. Such devices are relatively inexpensive, and are expected to become commonplace in laboratories using this type of data.

[9] Anderson R, Johnson GG, Bailey GW (1979). The Max-D Alphabetical Index to the JCPDS Database: A New Tool for Electron Diffraction Analysis.

